# Quantitative autofluorescence findings in patients undergoing hydroxychloroquine treatment

**DOI:** 10.1111/ceo.14090

**Published:** 2022-05-14

**Authors:** Salvatore Parrulli, Mariano Cozzi, Matteo Airaldi, Francesco Romano, Francesco Viola, Piercarlo Sarzi‐Puttini, Giovanni Staurenghi, Alessandro Invernizzi

**Affiliations:** ^1^ Eye Clinic, Department of Biomedical and Clinical Science “Luigi Sacco”, Sacco Hospital University of Milan Milan Italy; ^2^ Ophthalmological Unit Fondazione IRCCS Cà Granda Ospedale Maggiore Policlinico Milan Italy; ^3^ Department of Clinical Sciences and Community Health University of Milan Milan Italy; ^4^ Rheumatology Unit ASST‐Fatebenefratelli‐L. Sacco University Hospital Milan Italy; ^5^ The University of Sydney, Save Sight Institute, Discipline of Ophthalmology Sydney Medical School Sydney New South Wales Australia

**Keywords:** hydroxychloroquine, hydroxychloroquine retinopathy screening, multimodal imaging, quantitative autofluorescence

## Abstract

**Background:**

To measure quantitative autofluorescence (qAF) in patients under treatment with hydroxychloroquine (HCQ) and at risk of retinal toxicity but with no apparent signs of retinal toxicity and to compare it with that of untreated subjects.

**Methods:**

Consecutive patients at risk for the development of HCQ retinal toxicity (duration of treatment >5 years or daily HCQ dose >5 mg/kg of actual body weight [ABW]) but no alterations on spectral domain—optical coherence tomography, short‐wavelength autofluorescence and 10–2 visual field examination were recruited. Healthy subjects matched by age and sex were also enrolled in the study. All subjects underwent qAF measurements in one eye. Images were analysed using the conventional qAF grid by Delori calculating the qAF of eight sectors of the intermediate ring and the mean of those values (qAF_8_).

**Results:**

Thirty‐nine patients treated with HCQ (38 females, mean age 52.1 ± 8.6 years) and 39 untreated subjects (38 females, mean age 51.2 ± 8.6 years) were included. In both HCQ patients and untreated subjects, qAF_8_ was positively correlated with age (*p* = 0.004). Although HCQ patients showed a higher mean qAF_8_ compared with untreated subjects (294.7 ± 65.3 vs. 268.9 ± 57.5), the difference was not significant (*p* = 0.068). HCQ patients showed significantly higher mean qAF values in the inferior‐temporal, inferior and inferior‐nasal sectors of the intermediate ring of qAF grid compared with untreated subjects (all *p* < 0.05).

**Conclusions:**

These results suggest a possible preclinical increase of qAF values in inferior parafoveal sectors probably induced by HCQ exposure.

## 1 INTRODUCTION

Hydroxychloroquine (HCQ) is a relatively safe and effective drug widely used as primary or adjunctive treatment for several rheumatological and dermatological disorders.^1^, ^2^, ^3^ The employment of HCQ is rising due to its versatility, increasing clinical indications and few contraindications.^4^ HCQ modulates immune response through several mechanisms and has a tropism for pigmented ocular tissues, particularly retinal pigment epithelium (RPE).^5^ Its accumulation within RPE cells can lead to sight threatening retinal toxicity, with bull's eye maculopathy (BEM) and extended macular atrophy representing its advanced phenotype.^6^

Established risk factors for retinal toxicity consist of a daily dosage greater than 5 mg/kg of actual body weight (ABW), duration of therapy of more than 5 years, renal impairment or simultaneous tamoxifen use.^4^ For these reasons, ophthalmological screening is crucial in patients with these conditions in order to promptly diagnose HCQ‐related maculopathy and stop the drug intake to preserve retinal function.^7^

In 2016 the American Academy of Ophthalmologists (AAO) recommended optical coherence tomography (OCT) and short‐wavelength autofluorescence (SW‐AF) combined with 10–2 visual field (VF) testing as the tandard screening protocol, with multifocal‐electroretinography (mf‐ERG) being a useful tool in order to sort ambiguous cases out.^8^ Using these modalities, the prevalence of HCQ retinopathy (HCQR) is reported to be 4.7%–7.5% for treatment duration of more than 5 years.^9^, ^10^

**FIGURE 1:**
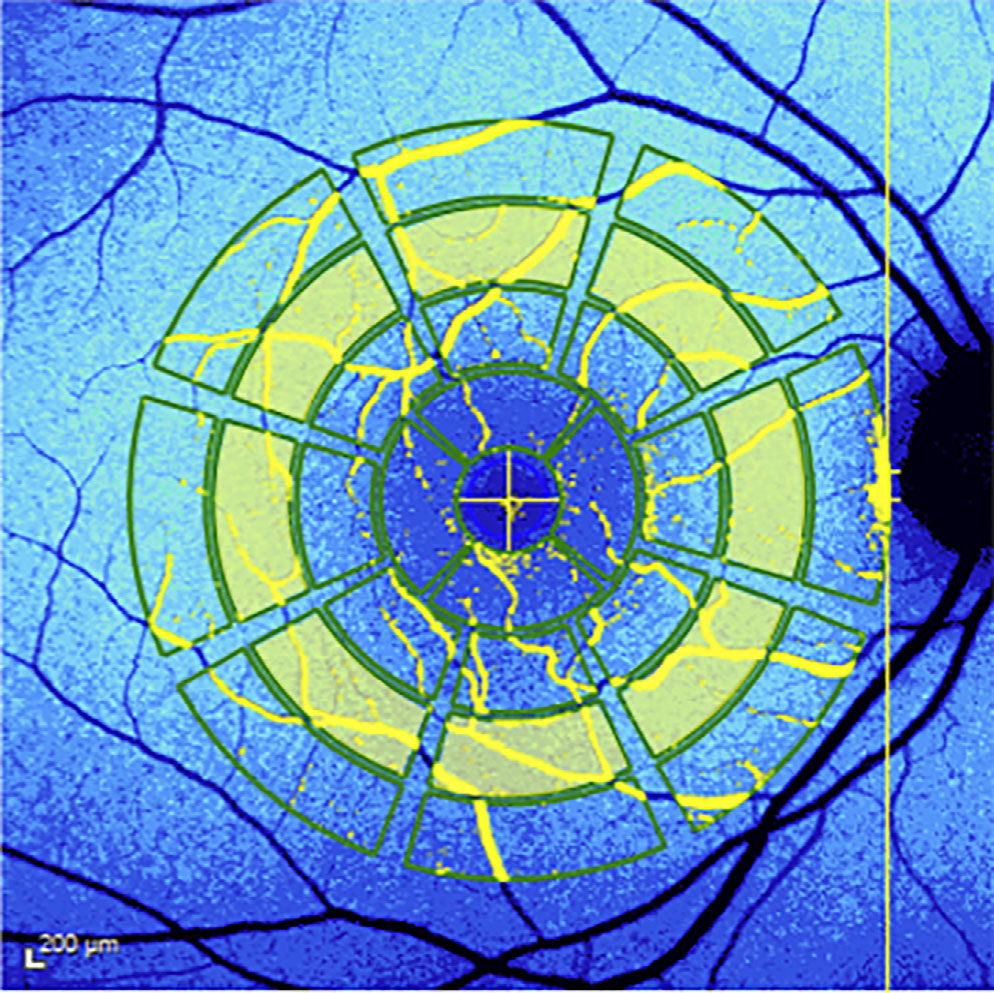
Colour‐coded quantitative Autofluorescence (qAF) map of a study patient. A Delori pattern grid has been superimposed and the eight subfields necessary to generate the qAF_8_ value have been highlighted in yellow

Quantitative autofluorescence (qAF) is an imaging modality that allows the measurement of retinal autofluorescence following short‐wavelength light (488 nm) excitation of bisretinoid fluorophores contained inside lipofuscin organelles.^11^ The normalisation of fundus autofluorescence signal gives the possibility to measure autofluorescence overtime in the same subjects and to compare results among different individuals.^11^, ^12^ Quantitative autofluorescence is thus based on a SW‐AF with a reference mirror installed, necessary to record and quantify the autofluorescence signal. For this reason, it is basically different from conventional SW‐AF. It is generally reported as qAF_8_, a parameter representing the mean qAF of eight segments constituting the middle ring of a specific grid adopted for qAF image analysis^13^ (Figure 1).

In healthy eyes qAF is known to increase with age and to correlate with retinal pigment epithelium/Bruch's membrane (RPE/BM) complex thickness.^14^, ^15^, ^16^ Altered levels of qAF have been observed in various macular diseases including Best vitelliform macular dystrophy, Stargardt disease, pattern dystrophies of the RPE and several other retinal conditions.^17^, ^18^, ^19^, ^20^, ^21^ Two recent studies have focused on qAF values in patients treated with HCQ.^22^, ^23^ In both studies qAF was increased in eyes with BEM. Furthermore, Reichel et al.^23^ were able to detect increased values of qAF in patients without BEM as early as 6 months after the start of HCQ treatment using an experimental imaging analysis procedure.

The aim of this study was to quantify the autofluorescence of patients treated with HCQ but with no signs of retinal toxicity according to standard screening guidelines using the conventional qAF_8_ grid and to compare it with that of age‐ and sex‐matched untreated subjects.

## 2 METHODS

This was a cross‐sectional study conducted at the Eye Clinic, Department of Biomedical and Clinical Science “Luigi Sacco” of Milan, University of Milan, Milan, Italy. The study was approved by the local Institutional Review Board (Milan Ethics Committee area 1). Informed consent was obtained from all participants at the time of enrollment. The study was conducted in adherence to the tenets of the Declaration of Helsinki.

### 2.1 Population

Patients under HCQ treatment followed at the Rheumatology Unit of L. Sacco University Hospital (Milan, Italy) or who were already regularly followed in our Eye Clinic for HCQ toxicity screening were consecutively recruited.

Inclusion criteria for enrollment were: age between 18 and 65 years, Caucasian ethnicity, best‐corrected visual acuity greater than 20/25, refractive error (spherical equivalent) between −3 and + 3 diopters, clear ocular media, no history of any ocular surgery including cataract extraction, duration of treatment with HCQ >5 years or duration of treatment <5 years with daily HCQ dose >5 mg/kg of actual body weight (ABW),^4^, ^8^ no sign of HCQ toxicity according to the results of the standard screening methods (SD‐OCT, SW‐AF, SITA 10–2 VF examination). Patients with macular diseases or retinal alterations (e.g., drusen) and patients taking other drugs with a known retinal toxicity (e.g., tamoxifen) were excluded in order to avoid interference in qAF evaluation.

A group of untreated subjects aged 18 to 65 years was also enrolled in the study. All of them were healthy volunteers of Luigi Sacco Hospital. Inclusion criteria for these subjects were as follows: best‐corrected visual acuity greater than 20/25, refractive error (spherical equivalent) between −3 and + 3 diopters, clear ocular media, no history of any ocular surgery including cataract extraction, absence of any ocular or systemic conditions that could affect the RPE physiology, and any previous or ongoing treatment known to cause RPE or retinal dysfunction. An extended although not complete list of these conditions is the following: blepharitis, dry eye syndrome, corneal opacities, uveitis, cataracts, vitreous opacities, asteroid hyalosis, age related macular degeneration, diabetic retinopathy, central serous chorioretinopathy, retinal vascular occlusion, retinal dystrophies (Best vitelliform macular dystrophy, Stargadt disease, retinits pigmentosa), choroidal dystrophies.

Among systemic conditions we consider all known diseases reported to potentially alter qAF: pseudoxantoma elasticum.

### 2.2 Image acquisition protocol

All the enrolled subjects underwent a complete ophthalmic examination including best‐corrected visual acuity (BCVA) using an ETDRS chart, biomicroscopy, intraocular pressure assessment, and funduscopic examination. Patients' eyes were dilated using 1% tropicamide and 2.5% phenylephrine eye drops. The examinator evaluated if the dilation was adequate in order to perform quantitative autofluorescence. The colour of the iris of each eye was categorised as brown or not brown (including blue, green or hazel) because the amount of pigment within the eye is known to possibly affect the qAF values.^14^

For each enrolled patient SD‐OCT scans and qAF images were acquired. Optical biometry was also performed to determine the corneal curvature using the Lenstar LS 900 (Haag‐Streit AG, Köniz, Switzerland). K1 and K2 keratometry values were averaged and transformed in order to determine the radius of corneal curvature value expressed in millimetres. This parameter is required to obtain reliable qAF and SD‐OCT measurements.^13^

Quantitative autofluorescence images were acquired as previously described by Delori et al.^13^ using a modified Heidelberg scanning laser ophthalmoscope (Spectralis HRA, Heidelberg Engineering, Heidelberg, Germany). In brief, the acquiring device was aligned to the pupil, and the 30 × 30° acquisition field was centred onto the fovea using a near‐infrared light. Photopigment was bleached by exposing the retina to an excitation light of 488 nm for at least 20 s. Multiple sets of 12 consecutive frames were then recorded in video format. At the end of the procedure, imaging quality was verified ensuring non‐flickering images, equal brightness between the frames, and no shadowing visible at the edge of each frame. To be considered acceptable, each sequence of 12 frames should have at least 9 frames meeting these quality criteria. The frames forming the videos were then averaged and saved without normalisation.^13^

A dense 30 × 25° SD‐OCT volume centred onto the fovea was acquired with a scan angle of zero degrees and using a Spectralis HRA2 (Heidelberg Engineering, Heidelberg, Germany). The entire pattern comprised 61 B‐scans with an inter B‐scan distance of 124 μm. Each single B‐scan was composed of 768 A‐scans, and the automated real time tracking (ART) average function was set to 16 frames. SD‐OCT and qAF images were acquired by a single expert operator (M.C.) in all cases.

### 2.3 Images analysis

One eye only from each subject was included in the analysis. For all subjects, the right eye was the elected study eye. If the right eye had poor‐quality images, the left eye was adopted for analysis. If both eyes had poor‐quality images, the subject was excluded.

Quantitative AF images were evaluated using the qAF analysis software provided by Heidelberg Engineering (qAF analysis software, V 6.0) and qAF values of eight subfields forming the intermediate ring centred onto the fovea (qAF_8_) and covering an area between 7 and 9° of eccentricity were collected. The OCT volume was essential in order to properly centre the Delori grid onto the fovea.

The exclusion of the central and external rings was previously proposed to avoid two main biases that could affect the quantitative measure of autofluorescence signal.^15^ In the central ring, the presence of the macular pigment that absorbs part of the blue light does not allow a reliable estimation of qAF.^24^ Similarly, the presence of large retinal vessels in the external ring could also affect the calculation of qAF values.^15^ The middle ring was therefore used for quantification as uniformity has been determined to be highest in this area.^11^ qAF_8_ measurements were calculated on the two best sets of images, and the average of the two sets was recorded to increase the accuracy, constituting the final qAF_8_ value for the intermediate ring. In order to enhance the accuracy of this measure, each qAF_8_ subfield was reviewed and excluded from the analysis in case of low‐quality signal. As previously described, the qAF formula accounts for age (lens transparency status) and corneal curvature to generate qAF values.^11^, ^14^

Spectral domain OCT images were analysed using the built‐in graph‐based automatic segmentation algorithm able to identify each retinal layer (Eye Explorer version 1.9.10.0, Heidelberg Engineering).

Each volume was manually double‐ checked to identify possible segmentation artefacts. When segmentation errors were identified in more than 1 B‐scan across the area of interest, the eye was excluded.

The software generates thickness maps of single retinal layers based on the Early Treatment Diabetic Retinopathy Study (ETDRS) grid. The map is formed by three concentric rings (central, inner, and outer), with the inner and outer ones further divided into four subfields each. For our analysis, RPE/BM complex and outer retinal layers (ORL) thickness values from the central, inner superior, inner temporal, inner inferior and inner nasal subfield were collected, since HCQ toxicity in Caucasians usually involves RPE and photoreceptors early on in the course of the disease, particularly in the parafoveal regions.^25^ On the automated segmentation of the Spectralis software, ORL is constituted by all layers from the ELM to the Bruch's membrane. Unfortunately, the ETDRS grid used to collect OCT‐based thickness values and the qAF_8_ grid do not exactly overlap.

### 2.4 Statistical analysis

Sample size calculation was performed considering a standard deviation of qAF_8_ values of 65.1, derived from a previously published paper on qAF_8_ in healthy eyes.^15^

Setting an effect size of 0.7, a type I error of 0.05 and a type II error of 0.8, we calculated that we would need 34 patients per group to observe a significant difference in the primary outcome.

Quantitative variables were analysed using the Shapiro–Wilk test in order to assess the normality of their distribution. Continuous variables were reported as mean (*SD*) or as median (interquartile range) when appropriate.

For categorical variables, statistical analysis was performed using Fisher's exact test or Pearson's Chi‐squared test as appropriate. For normally distributed continuous variables, a two‐sample *t* test was adopted.

Generalised linear mixed‐effects models were used to assess the influence of age, iris colour, gender and HCQ exposure (fixed effects) on both qAF_8_ and qAF values in different sectors. These models also included an identifier variable unique to each subject participating in the study as random effect. Estimated marginal means were employed to derive adjusted p‐values for both models.

All statistical analyses were performed using the open access software R version 4.0.0 (R Project—The R Foundation for Statistical Computing, Vienna, Austria). *p*‐values < 0.05 were considered statistically significant.

## 3 RESULTS

A total of 52 patients taking HCQ were screened from January 2018 to December 2018. Of those, 39 were eligible for our study and underwent quantitative AF measurements. Thirteen patients were excluded for poor quality images. Thirty‐nine age and sex‐matched untreated subjects were enrolled.

Mean age was 52.1 ± 8.6 years and 51.2 ± 8.6 years for HCQ patients and untreated subjects, respectively (*p* = 0.63). Thirty‐eight in the HCQ group and 38 in the untreated group were female (97.4% in both groups). A brown iris was present in 25/39 of HCQ patients (64.1%) versus 18/39 untreated subjects (46.2%), although the difference was not statistically significant (*p* = 0.13).

In the HCQ group, the median treatment duration was 13 years (interquartile range = 6–16.5 years), mean daily dose/ABW was 4.7 ± 1.4 mg/kg with a median cumulative dose of 1161.5 g (interquartile range 730.5–1625.4 g). Demographic characteristics are reported in Table 1.

**TABLE 1:** Demographic characteristics

	Case	Control
Number of eyes	39	39
Female	38 (97.4%)	38 (97.4%)
Age, years	52.1 (8.6)	51.2 (8.6)
Laterality OD	30 (76.9%)	23 (59%)
Iris brown	25 (64.1%)	18 (46.2%)
Iris other	13 (33.3%)	21 (53.8%)
qAF_8_	294.7 (65.3)	268.9 (57.5)
Weight, kg	64 (56.5, 70)	
Duration of intake, years	13 (6, 16.5)	
Daily dose/ABW, mg/kg	4.7 (1.4)	
Cumulative dose, g	1161.5 (730.5, 1625.4)	
**Indication for HCQ use**		
Systemic lupus erythematosus	4 (10.3%)	
Undifferentiated connective tissue disease	18 (46.2%)	
Rheumatoid arthritis	10 (25.6%)	
Sjögren's syndrome	7 (17.9%)	
Seronegative spondyloarthritis	1 (2.6%)	
Psoriatic arthritis	1 (2.6%)	

*Note*: Data are mean (*SD*), median (IQR) or *n* (%).

Abbreviations: ABW, actual body weight; HCQ, Hydroxychloroquine; qAF_,_ quantitative autofluorescence.

Mean ± *SD* qAF_8_ was 294.7 ± 65.3 and 268.9 ± 57.5 in the HCQ group and in the untreated group, respectively. The difference was not statistically significant (*p* = 0.068), even after adjusting for age, iris colour, gender and nesting of outcomes for subjects within matching pairs (*p* = 0.084). In the HCQ group, mean qAF_8_ was 295.3 ± 73.5 for patients with a daily dose >5 mg/kg and 268.8 ± 57.3 for patients with a daily dose <5 mg/kg. In addition, in this case the difference was not statistically significant (*p* = 0.23) (Table S1).

The analysis of each of the eight subfields of the intermediate ring of the qAF grid showed that the superior‐temporal sector was the one with the highest qAF values for HCQ patients. Similarly, the temporal, superior‐temporal and superior sectors showed higher qAF compared to other sectors in untreated subjects (Table 2). Interestingly, the HCQ group presented higher mean qAF values in all the inferior subfields (inferior‐temporal, inferior and inferior‐nasal) compared to untreated subjects, with differences being statistically significant also after adjusting the results for age, iris colour, gender and RPE thickness (see Table 2). No differences were found for the other sectors. A sub‐analysis of the HCQ group differentiating patients taking a daily dose of HCQ >5 or <5 mg/kg showed non‐significative differences in the qAF of the eight subfields (all *p* > 0.05).

**TABLE 2:** Values of qAF

	Middle ring qAF				RPE thickness (inner ring)			ORL thickness (inner ring)		
Location	HCQ mean (*SD*)	Controls mean (*SD*)	*p*‐value	Adjusted *p*‐value	HCQ mean (*SD*)	Controls mean (*SD*)	*p*‐value	HCQ mean (*SD*)	Controls mean (*SD*)	*p*‐value
Nasal	280.7 (65.5)	254.1 (57.4)	0.064	0.086	15.5 (1.6)	15.2 (1.6)	0.463	81.8 (3.2)	81.8 (3)	0.933
Superior‐nasal	300.2 (70.1)	281.4 (68.3)	0.232	0.199						
Superior	309.3 (66.7)	293.9 (73.8)	0.342	0.445	15.5 (1.9)	15.3 (1.6)	0.653	80.7 (3.2)	80.8 (3)	0.841
Superior‐temporal	323.9 (71.5)	293.8 (70.3)	0.068	0.058						
Temporal	316 (79.1)	295.1 (60.3)	0.247	0.061	15 (1.6)	14.7 (1.4)	0.359	81.4 (3.4)	81.3 (2.7)	0.899
Inferior‐temporal	288.6 (68.9)	256.9 (57.3)	**0.031**	**0.021**						
Inferior	267.6 (67)	231.3 (48.6)	**0.008**	**0.013**	15.1 (1.5)	14.6 (1.5)	0.189	79.8 (3.1)	79.4 (3.1)	0.529
Inferior‐nasal	278.5 (66)	247.5 (51.6)	**0.026**	**0.029**						
Central					16.7 (1.6)	16.9 (1.7)	0.702	89.4 (4.5)	88.9 (2.4)	0.598
Total	294.7 (65.3)	268.9 (57.5)	0.068	0.084	15.5 (1.4)	15.3 (1.3)	0.504	82.6 (3.2)	82.4 (2.6)	0.816

*Note*: Bold values are the statistical significant ones (*p*< 0.05).

RPE thickness and ORL thickness for each sector comparing HCQ patients and controls. *p*‐values adjusted for age, iris colour, gender.

Abbreviations: HCQ, hydroxychloroquine; ORL, outer retinal layers; qAF, quantitative autofluorescence; RPE, retinal pigment epithelium; *SD*, standard deviation.

The OCT analysis of the RPE and ORL thickness showed no differences between HCQ patients and untreated subjects (all *p* > 0.05) (Table 2). A generalised linear mixed model was adopted in order to obtain *p*‐values adjusted for age, iris colour, gender.

In both groups, mean qAF_8_ values were positively and significantly affected by age (*p* = 0.004) but not by iris pigmentation, and gender (*p* > 0.05) according to multiple regression analysis.

**FIGURE 2:**
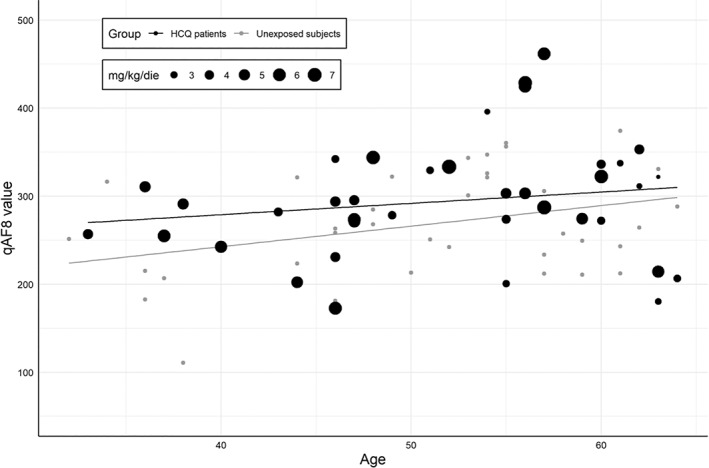
Graphic representation of qAF_8_ and role of age and HCQ daily dose/ABW. As known qAF_8_ increases with age with a logarithmic behaviour. Higher HCQ daily dosages are more often located above the middle line, although a clear correlation was not confirmed by multivariate analysis

With regards to the relationship between qAF_8_ values and HCQ exposition, neither daily dose/ABW, cumulative dose nor duration of therapy appeared to influence qAF_8_ in generalised linear mixed model regression. Measures of qAF_8_ plotted as a function of age and HCQ daily dose/ABW are reported in Figure 2.

**FIGURE 3:**
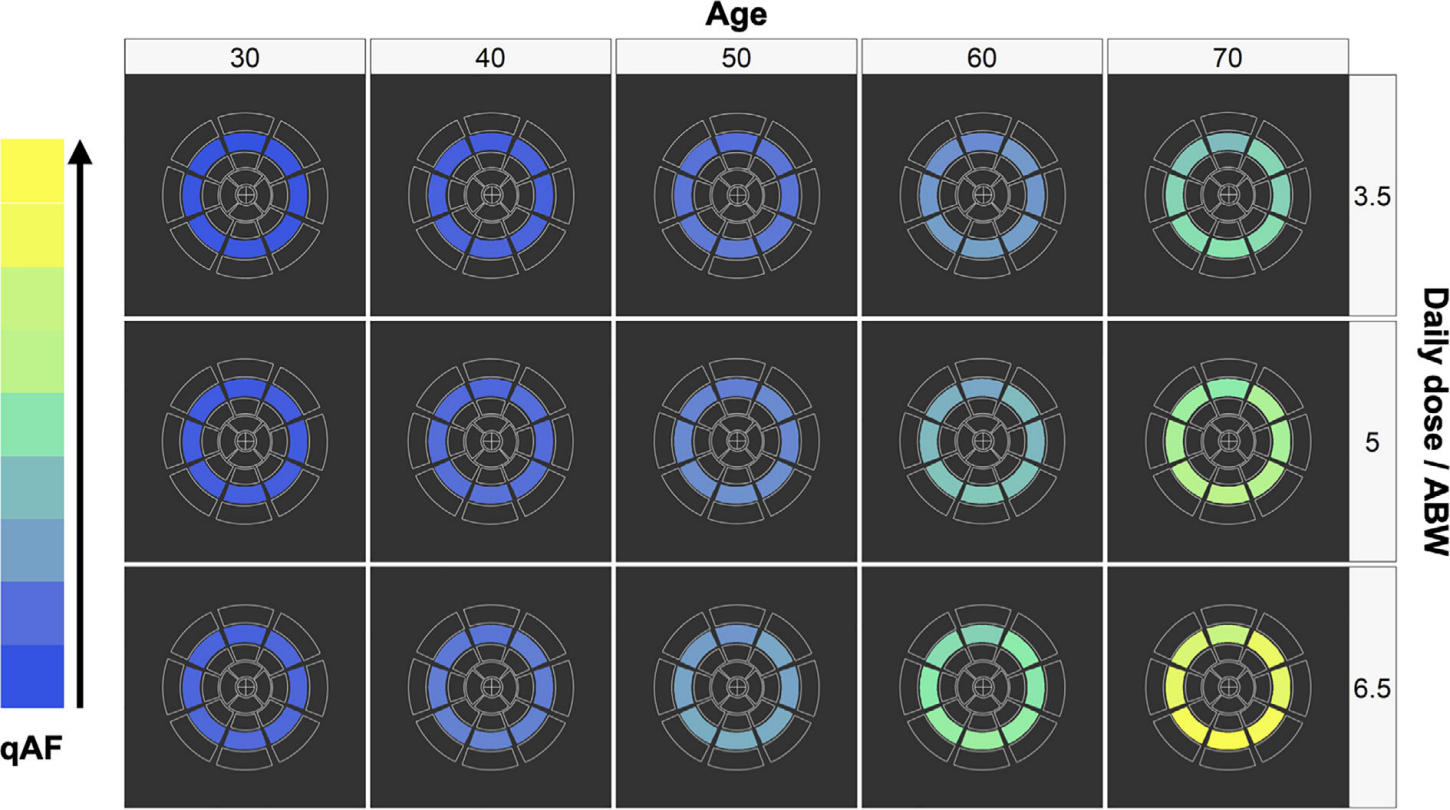
Visual representation of expected qAF values as influenced by age and HCQ daily dose/ABW, calculated for a treatment duration of 15 years. Expected values have been computed through GLMM regression (see methods section) and have been standardised for graphical purposes

Expected qAF values by middle ring sectors for different age and daily dose/ABW groups are graphically represented in Figure 3.

**FIGURE 4:**
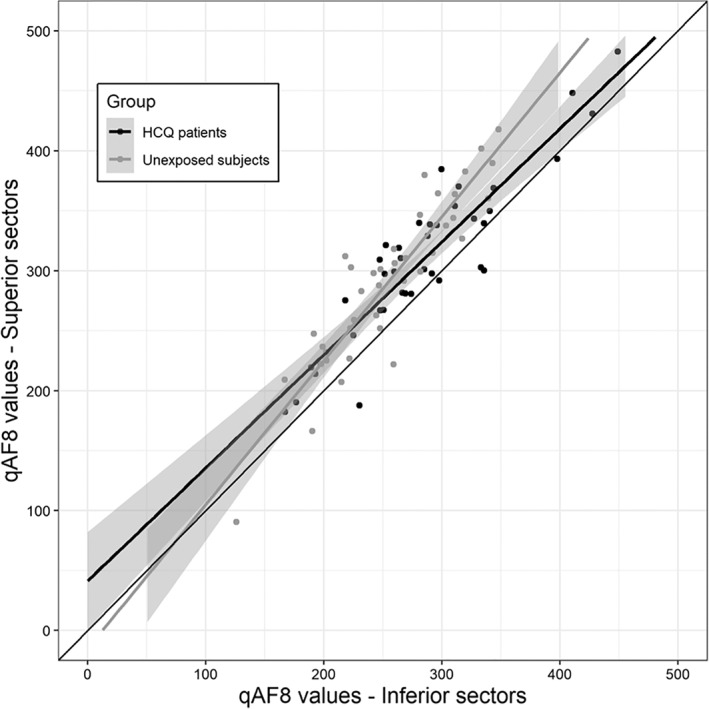
Scatter plot representing qAF values of the superior sectors (y axis) and of the inferior sectors (x axis). The slope of the trend line for unexposed patients appears greater compared to HCQ patients. This may be due to higher qAF values in the inferior sectors compared to superior sectors for HCQ patients

The mean ratio between the three superior and five inferior sectors was calculated and resulted greater for unexposed subjects compared to HCQ patients (1.14 ± 0.1 and 1.09 ± 0.1, *p* = 0.122). These results are summarised in a scattered plot (Figure 4).

## 4 DISCUSSION

In this study, we evaluated qAF in patients taking HCQ for more than 5 years and/or with daily HCQ dose >5 mg/kg of actual body weight (ABW) without functional or anatomical evidence of retinal toxicity according to standard screening methods and compared it with that of untreated subjects. Although the mean qAF_8_ did not appear to be significantly altered by the HCQ intake, the inferior sectors of macula showed a significant increase in qAF values in patients compared to untreated subjects. These results suggest that qAF could start increasing at a pre‐clinical stage in those areas of the macula which are known to be the first developing HCQ toxicity.^8^

There is still poor comprehension of preclinical retinal events associated with HCQ intake although early recognition of HCQ retino‐toxicity has improved in the last decade. Infact, subtle parafoveal abnormalities of outer retinal layers as well as inner and outer nuclear layers have been described as early OCT signs of retinal toxicity.^26^, ^27^ More importantly, once anatomical alterations become detectable by OCT or SW‐AF they are already irreversible and can even keep worsening after drug suspension.^4^ Identifying HCQ toxicity at a pre‐clinical stage would hence be ideal to prevent permanent vision loss. For this reason, we focused on patients without any evidence of retinopathy based on conventional screening procedures and we examined them with qAF.

In vitro, HCQ has been demonstrated to alter RPE lysosome pH, with consequent decrease of lysosomal lipofuscin degradation.^28^ This leads to a dramatic accumulation of undegraded lipofuscin material that accumulates inside RPE cells.^28^ Quantitative AF (qAF) offers a unique possibility to investigate RPE status indirectly through the evaluation of retinaldehyde‐adducts that mainly accumulate in its cells.^11^ Thus, this methodology should allow the detection of subtle metabolic alterations in the RPE when cells integrity is still preserved.

In our study qAF_8_ was significantly influenced by age in both treated and untreated subjects. This is in line with what reported both in healthy and diseased eyes.^13^, ^14^, ^15^, ^18^, ^19^, ^24^ Interestingly, we also found that qAF_8_ values were on average higher in HCQ patients than in untreated subjects, although this difference did not reach statistical significance. In addition, increased qAF_8_ values were not related to EZ/IZ thinning since all patients with detectable outer retinal layers alterations were excluded from the analysis.^26^, ^27^ These results are similar to those reported by Reichel et al.^23^ in a recent study where they evaluated the qAF in 29 patients taking HCQ with and without BEM. However, Reichel and colleagues used a specifically designed method for their measurements named qAF_97._ This approach is based on a 97‐sectors‐grid including macular regions arguably suitable for qAF measurement due to the higher density of retinal vessels.^15^ By contrast, we used the widely adopted qAF_8_ grid.^29^ For this reason, the results of the two studied cannot be directly compared.

In a recent study, Greenstein et al. quantified SW‐AF and near‐infrared autofluorescence (NIR‐AF) in 31 patients taking HCQ.^22^ The authors found an uneven distribution of the autofluorescence across the posterior pole with some regions showing an increased signal compared to others. They concluded that screening for HCQ retinopathy should take into consideration superior–inferior differences in susceptibility to HCQ toxicity. In our study, supero‐temporal and superior sectors were those with higher absolute qAF values both in HCQ patients and controls. This preferential distribution has previously been reported in healthy and diseased subjects.^11^, ^14^, ^15^, ^22^, ^23^ When compared to untreated subjects though, HCQ patients in our study had significantly higher qAF values in the infero‐temporal, inferior and infero‐nasal sectors. These regions are those initially affected by HCQ retinal toxicity,^4^, ^8^ with alterations of outer retinal layers and mottling of RPE detectable on OCT^26^ and subtle reduction of amplitudes on mf‐ERG.^30^ Since our patients had normal OCTs, SW‐AF and 10–2 VF it can be speculated that the increased qAF in these specific high‐risk areas may be expression of a pre‐clinical effect related to HCQ intake.

The reason why these areas of the macula are those primarily affected by HCQ toxicity is still unclear. Greenstein et al. speculated a possible role for the greater amount of incident light in inferior retinal regions.^22^ In fact, inferior sectors may be more exposed to free radical‐mediated apoptosis secondary to increased light exposure, as most light sources have an elevated location (sunlight and ceiling lightning).^31^ In this context, HCQ intake may predispose the macula to light toxicity, as it seems to be more severe in the inferior retina. A further role may be attributed to genetic predisposition in the development of HCQ retinopathy. ABCA4 genotype has already been postulated to play a role in HCQ retinal toxicity.^32^ In this regard, an ongoing clinical trial of the National Eye Institute (NEI) is trying to investigate any relationship between risk of HCQ retinopathy and ABCA4 variants.^22^

Solberg et al.^33^ evaluated HCQ patients performing fluorescence lifetime imaging ophthalmoscopy (FLIO). They observed a prolonged mean fluorescence lifetime in patients with HCQ retinopathy and normal mean values in HCQ patients without retinopathy compared to healthy subjects. More interestingly, Sauer et al.^34^ also detected a prolonged FLIO in patients exposed to HCQ with no signs of HCQ retinopathy, suggesting that this imaging modality may be useful in order to detect retinal toxicity before irreversible damage is manifest. A sectorial evaluation of fluorescence lifetime in inferior parafoveal quadrants may be useful in order to identify any localised alteration, not otherwise detectable calculating mean values of an inner and an outer ring. This approach may help to confirm our results.

This study has some limitations. Although we enrolled more subjects compared to previous studies on qAF in HCQ patients, our sample size could still be considered not enough to detect a significant effect of HCQ dosage on qAF. Due to its variance, qAF evaluation often requires a large number of enrolled subjects.^15^, ^35^, ^36^, ^37^ We speculated that the increased qAF identified in some macular areas of our HCQ patients could represent an early sign of qAF toxicity, but we could not confirm it due to the cross‐sectional nature of the study. A longitudinal evaluation with serial examination is needed to confirm the possible progression of the retinal damage to a clinically detectable stage. All patients but one were female, creating a consistent imbalance in our population. This distribution may be explained by the higher prevalence of rheumatological disorders requiring HCQ therapy among females.^38^ Finally the exclusion of patients with a low dosage HCQ intake lasting less than 5 years prevented us from verifying a possible early increase in qAF values.^23^ Furthermore, there is not an exact overlap between the subfields included in the qAF_8_ grid and the rings of the ETDRS. Therefore, we could not analyse the topographic correspondence between each single qAF value and the RPE/BM complex thickness in a specific subfield, but we calculated the correlation between the mean values of the two.

In conclusion, we observed increased qAF values in the inferior‐temporal, the inferior and the inferior‐nasal sectors in patients treated with HCQ without any evidence of retinal toxicity compared with untreated matched subjects. If confirmed, these results suggest a possible role of the qAF for the identification of HCQ retinal toxicity at a pre‐clinical stage in a large cohort of patients.

## CONFLICT OF INTEREST

Salvatore Parrulli, no financial disclosures; Mariano Cozzi, Recipient: Bayer, Nidek, Zeiss; Matteo Airaldi, no financial disclosures; Francesco Romano, no financial disclosures; Francesco Viola, Novartis (C), Bayer (C), Roche (C); Piercarlo Sarzi‐Puttini, no financial disclosures; Giovanni Staurenghi, Heidelberg Engineering (C), QuantelMedical (C), Centervue (C), Carl Zeiss Meditec (C), Alcon (C), Allergan (C), Bayer (C), Boheringer (C), Genentech (C), GSK (C), Novartis (C), and Roche (C), Optos (F), Optovue (F) and Centervue (F); Alessandro Invernizzi, Novartis (C), Bayer (C).

## Supporting information


**Supplementary Table 1** Detailed case group characteristics
