# Supplementary material for: Quantitative autofluorescence findings in patients undergoing hydroxychloroquine treatment
Source: Clin Exp Ophthalmol. 2022 May 14;50(5):500–9. doi: 10.1111/ceo.14090 (PMC9545387; doi:10.1111/ceo.14090)
Supplement: Supplementary file 1 — Supplementary Table 1 Detailed case group characteristics [file CEO-50-500-s001.docx]

| **Supplementary Table 1.** Detailed case group characteristics | | | | | | | | | | |
| --- | --- | --- | --- | --- | --- | --- | --- | --- | --- | --- |
| **n°** | **Age (y)** | **Sex** | **Indication for HCQ**†  **use** | | **Included eye** | **Weight**  **(kg)** | **Daily dose/ABW**¶¶  **(mg/kg)** | **Duration of intake (y)** | **Cumulative dose (g)** | **qAF_8_** ††† |
| **1** | 33 | F | SLE‡ | | right | 69 | 4.35 | 15 | 1642.5 | 257.01 |
| **2** | 36 | F | UCTD § | | left | 52 | 5.00 | 5 | 474.5 | 310.69 |
| **3** | 37 | F | UCTD | | right | 66 | 6.06 | 12 | 1752.0 | 254.86 |
| **4** | 38 | F | UCTD | | right | 80 | 5.00 | 5 | 730.0 | 291.12 |
| **5** | 40 | F | RA ¶ | | right | 52 | 5.77 | 5 | 547.5 | 242.54 |
| **6** | 43 | F | RA | | right | 53 | 3.77 | 8 | 584.0 | 282.06 |
| **7** | 44 | F | SLE + SjS | | right | 59 | 5.25 | 5 | 565.8 | 202.22 |
| **8** | 46 | F | UCTD | | right | 44 | 4.55 | 20 | 1460.0 | 293.85 |
| **9** | 46 | F | SLE | | right | 64 | 6.25 | 17 | 2482.0 | 172.88 |
| **10** | 46 | F | UCTD | | right | 94 | 4.26 | 15 | 2190.0 | 230.98 |
| **11** | 46 | F | UCTD | | right | 60 | 3.33 | 24 | 1752.0 | 342.34 |
| **12** | 47 | F | RA | | right | 55 | 5.45 | 6 | 657.0 | 271.15 |
| **13** | 47 | F | RA | | right | 49 | 6.12 | 20 | 2190.0 | 273.49 |
| **14** | 47 | F | SjS †† | | left | 68 | 4.41 | 9 | 985.5 | 295.42 |
| **15** | 48 | F | SjS | | right | 60 | 6.67 | 17 | 2482.0 | 344.08 |
| **16** | 49 | F | RA | | right | 58 | 3.45 | 18 | 1314.0 | 278.37 |
| **17** | 51 | F | UCTD | | right | 62 | 3.23 | 15 | 1095.0 | 329.49 |
| **18** | 52 | F | SLE | | right | 54 | 7.41 | 17 | 2482.0 | 333.30 |
| **19** | 54 | F | RA | | right | 150 | 2.67 | 15 | 2190.0 | 396.09 |
| **20** | 55 | F | RA | | right | 64 | 3.13 | 17 | 1241.0 | 200.86 |
| **21** | 55 | F | UCTD | | right | 54 | 3.70 | 6 | 438.0 | 273.91 |
| **22** | 55 | F | SjS | | left | 43 | 4.65 | 22 | 1606.0 | 303.07 |
| **23** | 56 | F | UCTD | | right | 75 | 5.33 | 10 | 1460.0 | 303.30 |
| **24** | 56 | F | SpA ‡‡ | | left | 65 | 6.15 | 5 | 730.0 | 424.76 |
| **25** | 56 | F | RA | | right | 60 | 6.67 | 6 | 876.0 | 428.96 |
| **26** | 57 | F | UCTD | | right | 65 | 6.15 | 13 | 1898.0 | 461.72 |
| **27** | 57 | F | UCTD | | left | 85 | 7.06 | 6 | 1314.0 | 287.32 |
| **28** | 59 | F | SjS + RA | | left | 70 | 3.03 | 15 | 1160.7 | 276.46 |
| **29** | 59 | F | UCTD | | right | 74 | 5.41 | 6 | 876.0 | 274.53 |
| **30** | 60 | F | UCTD | | right | 59 | 3.39 | 13 | 949.0 | 272.16 |
| **31** | 60 | F | SjS | | right | 52 | 3.85 | 15 | 1095.0 | 336.14 |
| **32** | 60 | F | PA §§ | | right | 60 | 6.67 | 7 | 1022.0 | 322.38 |
| **33** | 61 | F | SjS | | left | 70 | 2.86 | 16 | 1168.0 | 337.33 |
| **34** | 62 | F | UCTD | | left | 75 | 2.67 | 18 | 1314.0 | 311.53 |
| **35** | 62 | F | UCTD | | left | 70 | 4.29 | 6 | 657.0 | 353.34 |
| **36** | 63 | F | UCTD | | right | 70 | 2.86 | 16 | 1168.0 | 180.43 |
| **37** | 63 | F | RA | | right | 85 | 2.35 | 8 | 584.0 | 321.76 |
| **38** | 63 | M | UCTD | | right | 70 | 5.71 | 5 | 730.0 | 214.32 |
| **39** | 64 | F | UCTD | | right | 62 | 3.23 | 6 | 438.0 | 206.64 |
| † Hydroxychloroquine; ‡ systemic lupus erythematosus; § undifferentiated connective tissue disease; ¶ rheumatoid arthritis; †† Sjögren's syndrome; ‡‡ Seronegative spondyloarthritis; § § Psoriatic arthritis; ¶¶ actual body weight; ††† quantitative autofluorescence | | | | | | | | | | |
|  |  |  | |  |  |  |  |  |  |  |
